# Do differences exist in the weight of fetus, placenta and umbilical cord in normal pregnant women and in well controlled gestational diabetic pregnant women?

**DOI:** 10.1186/1758-5996-7-S1-A72

**Published:** 2015-11-11

**Authors:** Franciele Pereira Madeira, Edson Nunes de Morais, Tatiana Frehner Kovalco, Francisco Maximiliano Pancich Galarretta, Caren Leivas Pozzer, Walter Santos Neme, Karina Soares

**Affiliations:** 1 Universidade Federal de Santa Maria, Santa Maria, Brazil

## Background

The weight of fetus, placenta and umbilical cord are important parameters that can reflect the environment where the fetus will grow. We analysed these parameters in well controlled gestational diabetic pregnant women (GDP) and compare this data with normal pregnant women (NG) to reveal the importance of this regulation.

## Objectives

The present search wants to show the importance of the control of the glicemia levels in pregnant women that develop GDP based on parameters that can reflect the environment where the fetus will grow.

## Materials and methods

The weight of fetus, placenta and umbilical cord were measured in 30 (thirty) normal pregnant women and in 17 (seventeen) controlled gestational diabetic pregnant women met in HUSM (University Hospital of Santa Maria). The measurements of weight of fetus were made immediately after the delivery and the macroscopy exam of placenta and umbilical cord were made 24 (twenty four) h after the delivery using a precision scale. A statistical analysis was made using SPSS 17 with Mann Whitney Test considering significant p values <0.05.

## Results

Fetal, placental and umbilical cord medium weights obtained first in NG and after in GDP in the macroscopy exam were respectively: 3.387g±405g and 3.633g±580g; 496g±93g and 559g±171g; 34g±14g and 48g±19g. The statistical analysis showed no significant difference between the values obtained of NG and GDP.

## Conclusion

The results allowed us to conclude that fetal, placental and umbilical cord weight studied in NG and in GDP have measures not significantly different. These results show the importance of pre natal control of the alterations that GDP can have in metabolism. Although we can see a tendency of higher parameters in GDP, these numbers don't significantly affect the growth of the fetus in well controlled GDP.

